# Structural Determinants of Health among Im/Migrants in the Indoor Sex Industry: Experiences of Workers and Managers/Owners in Metropolitan Vancouver

**DOI:** 10.1371/journal.pone.0170642

**Published:** 2017-01-31

**Authors:** Shira M. Goldenberg, Andrea Krüsi, Emma Zhang, Jill Chettiar, Kate Shannon

**Affiliations:** 1 Gender and Sexual Health Initiative, British Columbia Centre for Excellence in HIV/AIDS, Vancouver, B.C., Canada; 2 Faculty of Health Sciences, Simon Fraser University, 8888 University Drive, Burnaby, BC, Canada; 3 Division of AIDS, Department of Medicine, University of British Columbia, Vancouver, B.C., Canada; Agencia de Salut Publica de Barcelona, SPAIN

## Abstract

**Background:**

Globally, im/migrant women are overrepresented in the sex industry and experience disproportionate health inequities. Despite evidence that the health impacts of migration may vary according to the timing and stage of migration (e.g., early arrival vs. long-term migration), limited evidence exists regarding social and structural determinants of health across different stages of migration, especially among im/migrants engaged in sex work. Our aim was to describe and analyze the evolving social and structural determinants of health and safety across the arrival and settlement process for im/migrants in the indoor sex industry.

**Methods:**

We analyzed qualitative interviews conducted with 44 im/migrant sex workers and managers/owners working in indoor sex establishments (e.g., massage parlours, micro-brothels) in Metropolitan Vancouver, Canada in 2011; quantitative data from AESHA, a larger community-based cohort, were used to describe socio-demographic and social and structural characteristics of im/migrant sex workers.

**Results:**

Based on quantitative data among 198 im/migrant workers in AESHA, 78.3% were Chinese-born, the median duration in Canada was 6 years, and most (86.4%) serviced clients in formal indoor establishments. Qualitative narratives revealed diverse pathways into sex work upon arrival to Canada, including language barriers to conventional labour markets and the higher pay and relative flexibility of sex work. Once engaged in sex work, fear associated with police raids (e.g., immigration concerns, sex work disclosure) and language barriers to sexual negotiation and health, social and legal supports posed pervasive challenges to health, safety and human rights during long-term settlement in Canada.

**Conclusions:**

Findings highlight the critical influences of criminalization, language barriers, and stigma and discrimination related to sex work and im/migrant status in shaping occupational health and safety for im/migrants engaged in sex work. Interventions and policy reforms that emphasize human rights and occupational health are needed to promote health and wellbeing across the arrival and settlement process.

## Background

Global estimates suggest that there are approximately 232 million international migrants worldwide, of whom approximately half are women[1]. Stemming from intersecting social and structural inequities including gender, economic marginalization, and racialization, immigrant and migrant women in destination settings often fill low-paying positions in the informal sector, where they often face insecurity and unsafe working conditions, including lower access to occupational health and safety[2–4]. Although women represent over half of migrants globally, there remains a paucity of information regarding the gender-based impacts of migration on women’s health and safety, particularly among those working in the sex industry. As with other populations, immigrant and migrant (im/migrant) women most commonly become involved in sex work for economic (e.g., subsistence needs, remittances) reasons, with the sex industry as one of few informal sectors in which employment in a new destination country may be easier to obtain[5–9].

Although a growing body of research has highlighted the enhanced vulnerabilities of female sex workers to HIV, sexual and reproductive health problems[10–13] and human rights violations, including violence[14–17], limited information is available regarding the health and working conditions of im/migrants in the sex industry. Despite the significant number of women who migrate, much research pertaining to the health and safety of mobile and migrant workers in the formal and informal sectors has focused on males, including resource-extraction workers, farmworkers, and truck drivers[18–21], and comparatively less attention has been paid to the health and social needs of female im/migrant migrant labourers in destination contexts. In this analysis, the term im/migrant is used to refer to individuals who leave their country of origin or of habitual residence, to establish themselves in another country, and includes those with and without legal immigration status (e.g., permanent residents, temporary workers, undocumented).

Previous research in destination countries has shown that migration can result in substantial changes in health, working conditions, and economic and social opportunities. Upon arrival in a new destination, im/migrants may experience changes in *health status* related to exposure to new infectious diseases, social networks (e.g., new sex partners), norms (e.g., regarding drug use), and barriers to accessing healthcare.[6, 20, 22–29] They are also likely to experience *new working conditions*, and have been shown to be more likely to work in unsafe work environments, compared to non-migrant populations[30, 31]. Im/migration may also lead to *social and economic changes*, such as exposure to discrimination[32, 33] or enhanced socio-economic marginalization, particularly among more marginalized im/migrant groups[20, 23, 34]. At the same time, moving to a new country can improve access to higher-paying work and new employment opportunities, particularly for individuals whose employment options may have been more restricted in home communities.

Evidence indicates that to better understand the complex relationship between migration and health, there is a need to consider how the social and structural determinants im/migrant populations are exposed to vary by the timing, duration, and phase of migration (e.g., pre-migration, migration/transit, arrival, long-term settlement in destination settings).[35] Previous research has often attributed documented changes in the health of im/migrant populations over time to acculturation (i.e., the gradual adoption of social and cultural characteristics and habits of the host setting), with most of this research having been conducted in the U.S.[36, 37]

Migrant and immigrant health researchers have increasingly advocated for social epidemiological and qualitative research that acknowledges the impacts of the social, political, and economic contexts within which im/migrants are embedded on health inequalities. For example, differential exposure to social and structural determinants, such as unequal access to economic and social opportunities, and experiences of discrimination and racialization[26, 33], have been identified as key areas for future research. Drawing on these theoretical foundations, this research aimed to explore the ways in which changing exposure to social and structural determinants (e.g., discrimination, unequal access to healthy living and working opportunities) across the migration process influence the health and wellbeing of marginalized im/migrant women.[26, 32, 33]

This analysis draws on a socio-ecological conceptualization of health and wellbeing, which emphasizes that health is an outcome of social and structural conditions and, in particular, sociocultural, economic and political inequalities[38, 39]. This approach highlights the interconnectedness of the biological with social, political, legal and economic forces, to move beyond individually focused and behavioural approaches to understanding health and wellbeing. Our analysis brings together conceptual theories related to the social and structural determinants of im/migrant[26] and sex workers’ health[40], including conceptualizations of structural vulnerability and violence[41–43] which draw attention to the ways in which unequal power and life chances shape the health, safety and wellbeing of marginalized populations[44]. These social and structural determinants are shaped by broader intersecting macro-structural forces (e.g., gender inequities, criminalization of sex work, restrictive immigration policies, poverty, racism) which are rooted in historical and economic processes (e.g., colonialism, globalization).

Given relatively limited scholarly attention to social and structural determinants of migrant health and wellbeing across the migration process, this study aimed to describe and analyze the evolving social and structural determinants of health and safety across the arrival and settlement process for im/migrants in the indoor sex industry in Metropolitan Vancouver, Canada.

## Methods

### Study setting

This study was conducted in Metropolitan Vancouver, Canada. Canada is the second largest destination country for immigrants in the Americas, with immigrants comprising over 20% of Canada’s population (the highest proportion among G8 countries).[45] Within Metropolitan Vancouver, over 50% of the population are immigrants[46], the majority of whom are from Asia.[47] In Canada, formal indoor sex establishments are diverse and operate as licensed body rub studios, massage parlours, spas, beauty parlours, and health enhancement/wellness centres, as well as unlicensed micro-brothels (i.e., private home/apartment where two or more workers operate). Although no reliable statistics exist on the number of im/migrant sex workers in Vancouver, a significant proportion of sex workers are believed to be foreign-born[48], including most of those in formal indoor establishments. In Canada, most aspects of sex industry work have been historically criminalized, including public solicitation, running a ‘bawdy house’, and ‘living on the avails of prostitution’. Following a 2013 Supreme Court ruling which deemed the previous laws to be unconstitutional, new legislation was introduced in 2014 (*Bill C-36*: *The Protection of Exploited Persons and Communities Act)* criminalizing the purchasing of sex, solicitation/communication in public areas where minors may be present, and third parties who advertise for and receive financial or material benefit from sex work[49].

### Ethical considerations

All participants provided written informed consent prior to participating in the study, which was obtained by trained multilingual female interviewers. The study protocol and consent procedures were approved by the Providence Healthcare/University of British of Colombia Research Ethics Board.

### Qualitative data collection

In 2011, in-depth interviews were conducted with im/migrant workers and managers/owners in indoor sex establishments (e.g., massage parlours/body rub studios, health enhancement centres, micro-brothels) across Metropolitan Vancouver, as previously described[50].

Participants were recruited through intensive outreach to diverse indoor sex work venues (e.g., massage parlours/body rub studios, health enhancement centres, micro-brothels) as well as online. Recruitment was conducted during regular weekly outreach (e.g., condom distribution, health education) visits to indoor and online sex work spaces by a multilingual and multicultural outreach team with extensive sex work community experience.

Eligible participants were women aged 18 years or older, im/migrant to Canada (i.e., born outside Canada) who worked in an indoor sex establishment within the last 30 days as a worker (exchanged sex for money in the last month), manager/owner or both. Participants were purposively selected to reflect a range of worker and manager/owner experiences, representing differences in types of indoor establishments (e.g., massage parlour/body rub studio, acupressure, health enhancement centre, micro-brothel) and duration in Canada.

The present analysis included in-depth interviews conducted with 44 im/migrant sex workers and managers/owners of Chinese nationality. Of the 44 participants, the median age was 42 years old. Participants were Chinese-born in/migrants who had lived in Canada for a median of 7 years. Most identified as sex workers (n = 39), over half as managers/owners (n = 23), and 15 worked in both roles. Most operated in licensed establishments such as health enhancement centres (n = 18) or body rub parlours (n = 19), and the remainder worked in other types of establishments (e.g., unlicensed establishments such as micro-brothels, acupressure establishments)

Multilingual female interviewers with extensive sex work community experience conducted the interviews in private storefront offices or in the confidential location of participants’ choosing (e.g., workplace, home). Interviews were held in English or Mandarin, lasted between 30 and 120 minutes and were audio-recorded. All participants received CAD $30 for their time and expertise.

The interviews were based on a loosely structured interview guide that was pilot-tested and revised prior to implementation. Questions were designed to elicit narratives on participants’ im/migration histories (e.g., moving to Canada, socio-economic challenges upon arrival), im/migration-related barriers (e.g., language skills and barriers, cultural differences, discrimination), drivers of sex work involvement, and ongoing health and social challenges, including condom negotiation, and interactions with police, immigration, and city officials. Interviews were transcribed verbatim and translated into English by trained, bilingual research assistants.

### Quantitative data collection

This project complemented an ongoing community-based cohort study of street and off-street sex workers across Metropolitan Vancouver, Canada, known as AESHA (An Evaluation of Sex Workers Health Access). AESHA is guided by a Community Advisory Board of over 15 community sex work, health, and social service organizations, as previously described.[51] Between 2010–2013, 722 female sex workers who were ≥ 14 years old, exchanged sex for money within the last month, and were able to provide written informed consent completed quantitative surveys and serological testing for HIV/STIs, as previously described[52]. Of these, 198 (27.4%) were im/migrants born outside of Canada. Im/migrants were recruited through multilingual outreach to indoor establishments (e.g., massage parlours, micro-brothels) and online. Participants completed semi-annual questionnaires in English, Mandarin or Cantonese administered by trained interviewers and HIV/STI testing by a trained project nurse.

### Data analysis

Qualitative interviews were coded in Atlas.ti 7 and systematically analyzed for thematic content. Our team of academic and community team members discussed the content of interviews, emerging themes, and coding framework throughout the data collection and analytic processes. Coding was conducted by both academic and community-based team members, including multilingual/multicultural interviewers with extensive sex work community experience, and a sex worker participant/co-author provided key community input on the results and their interpretation. The current analysis was restricted to Chinese-born participants (n = 44) and began by coding the transcripts for key themes that emerged in the data, including themes related to arrival and settlement in Canada (e.g., immigration to Canada, language barriers, cultural issues, barriers to employment), health and safety (e.g., barriers to condom negotiation, workplace violence), legal concerns (e.g., interactions with police, immigration, and municipal inspectors; licensing requirements; criminalization). Major analytic decisions were discussed as a team, and a codebook was maintained. Key themes were grouped and regrouped until a set of themes that described the main social and structural determinants of health reflected in participants’ narratives across the arrival and settlement process was generated. The results presented under each category reflect the themes and sub-themes identified by our analysis (i.e., coding) of the interviews.

To supplement and contextualize our analysis of the qualitative data, we analysed and drew upon key quantitative data derived from the questionnaire responses of im/migrants participating in the larger AESHA cohort. Using SAS Version 9.1, descriptive statistics were calculated in to describe socio-demographic and social and structural characteristics of im/migrant sex workers in the AESHA cohort (Table 1).

**Table 1:** Quantitative baseline characteristics of im/migrant sex workers in AESHA, Metropolitan Vancouver, Canada, 2010–2013 (*n =* 198).

Characteristic	n (%) (n = 198)
*Socio-demographics*	
Age, in years (median and interquartile range, IQR)	37 (30–42)
Born in China	155 (78.3%)
Duration in Canada (median, IQR)	6 (3–11)
Duration in sex industry, in years (median, IQR)	2 (0–5)
*Social and structural determinants*	
Place of servicing clients	
• Formal indoor establishment	6.4%)
• Informal indoor establishment	5.1%)
• Outdoor/public space	17 (8.6%)
Educational attainment: High school completion or greater	165 (83.3%)
Financially supports dependents	113 (57.1%)
Language most comfortable speaking	
• Mandarin	7.7%)
• English	15.2%)
• Cantonese	23 (11.6%)
Sex industry as main source of income	182 (91.9%)
Average monthly income, in CAD (median, IQR)	$3200 (2000–5724)
Experienced police harassment or arrest	42 (21.2%)
Client-perpetrated physical/sexual violence	33 (16.7%)

NOTE: All values represent n (%) of participants, unless otherwise noted.

## Results

### Socio-demographic characteristics and social and structural factors among im/migrant sex workers

Of the 198 im/migrant workers enrolled in AESHA, most were Chinese-born (*n =* 155, 78.3%) and the median duration in Canada was 6 years (IQR: 3–11). Most had high school level education (*n =* 165, 83.3%) or greater. The median duration in sex work was 2 years (IQR: 0–5), and the median number of clients serviced monthly was 40 (IQR: 24–60). Most im/migrant workers serviced clients in formal indoor venues (e.g., licensed health enhancement centres, body rub studios) (*n =* 171, 86.4%).

### Findings of thematic analysis

#### Arrival in Canada and diversity of pathways into sex work

Migration and arrival to Canada: Analysis of qualitative interviews revealed that participants typically came to Canada legally in search of improved economic opportunities, and contrary to popular belief, none described having been trafficked. AESHA data indicate that the majority of im/migrant workers financially support dependents (*n =* 113, 57.1%), primarily children (40.9%) and parents (21.2%). Im/migrant workers often described barriers to traditional labour markets, combined with economic pressures, as the primary challenges they faced upon initial arrival to Canada:

**Although I am a new immigrant coming to a country formed by immigrants, it still takes a process to adapt.** It is not as easy as you would think…I didn’t have a specific skill to sell, my limitations make me easily replaceable, and the pay rate was low. It is hard to get by with low pay jobs, as the living cost in Canada is quite high and **I have other sources of pressure such as family members to support.**

[QE38, worker, 4y in Canada]

#### “Language was a big barrier”: Language barriers to conventional labour markets

Although participants typically arrived with some English skills, limited English fluency made it difficult, if not impossible, for most to find work upon arrival. Of im/migrants in AESHA, most reported being most comfortable speaking Mandarin (*n =* 134, 67.7%), followed by English (*n =* 30, 15.2%) and Cantonese (*n =* 23, 11.6%). Participants frequently discussed the language barriers they faced to conventional labour markets during the initial arrival period:

We do not have the country’s official language skills to work. Even after filling out a lot of applications, no one would acknowledge you. I even applied to Chinese-run businesses and did not get any responses. **The first three months were of long suffering**; I felt that I was a useless person.

[QE15, worker, 10y in Canada]

The language barrier really is difficult to overcome…For many jobs, you need to have in-depth communication with people, so you need to have very proficient language skills, but **we are not able to achieve that**…It is a very big obstacle…**it restricts our entire life.**

[QE13, manager/worker, 2y in Canada]

Language was a barrier…I only knew Mandarin. My job selection was quite limited…I needed income to cover living costs. **I wasn’t in a position to wait until my English skills improved, I needed to work.**

[QE44, worker, 1y in Canada]

#### Limited recognition of foreign education

Language barriers intersected with limited recognition of credentials received overseas to exacerbate employment barriers for many women. As a worker who had been living in Canada for eight years noted, “We don’t have the language skills or the required education levels. We all had good jobs in China…but now we all have to start from scratch.” Although most participants expressed a desire to return to school to improve their English or update their professional qualifications, they emphasized the economic and practical challenges they faced in doing so while struggling to get by as a new immigrant:

Th**e professional status we received or achieved in China are not recognized here.** Our diplomas and certificates are not recognized here…We have to face many different obstacles.

[QE30, manager, 10y in Canada]

The biggest challenge was that I didn’t have a degree…our Chinese educations are not recognized here…if **I had money, I didn’t need to worry about housing, and I had enough to support my children, then I might go to school**…but my kids are young, and financially there is a lot of stress.

[QE20, worker, 10y in Canada]

#### Limited social networks in destination country

Limited access to social networks and support in Canada also influenced the challenges faced by recent arrivals to Canada. Many participants reflected on the implications of a lack of social support to buffer the dislocating effects of moving to a new country. Importantly, not having social networks that could be relied upon for economic support (e.g., a loan, a place to stay) or to connect recent im/migrants to better-paying jobs were described as critical vulnerabilities faced by new immigrants:

**It is very difficult for us, especially when you first get to Canada,** after half a year, you spent all the money you brought, and you have to find work, and it’s so hard. **Also you don’t have any social networks here, you can’t rely on anyone.**

[QE33, worker, 8y in Canada]

If you don't know anyone to refer you [to a better job], you wouldn't be able to get in on your own…**But I didn't know anyone with these kinds of relationships**…we went to the government or those professional job referral centres, but we just waited a long time and didn't get any jobs either…**For new immigrants, this is a big issue.**

[QE25, manager/worker, 5y in Canada]

#### Sex work as a higher-paying and flexible employment opportunity post-arrival

Upon initial arrival in Canada, most participants worked in minimum-wage jobs such as manufacturing, supermarket work, or waitressing, and described their earnings as grossly insufficient to meet the high cost of living in Vancouver. As one participant put it, “getting paid $8 or $10 an hour is not enough for eating and surviving” (QE21).

Participants in this qualitative study all described entering sex work consensually following arrival in Canada. Most im/migrants in AESHA reported initiating sex work within the prior two years, and that this comprised the primary income source of most participants (*n =* 182, 91.9%). All participants’ initial experiences in the sex industry occurred following arrival in Canada, and importantly, none reported having moved to Canada or entering the sex industry due to trafficking or other forms of force/coercion. Rather, interviewees contextualized their involvement in the sex industry as a response to their economic and family needs upon moving and settling in Canada, in combination with the higher pay and more flexible working conditions (e.g., flexible hours to accommodate childcare) characterizing sex work:

I tried to do other jobs, but the money I made wasn’t enough…I worked as a tutor, bank teller…I didn’t get off the plane and immediately went to work as a sex worker. **I was searching around for a good job and was at a loss for several years, and then I found out people made money at massage parlours.**

[QE32, worker, 8y in Canada]

Sex work was often framed as a comparatively high-paying and flexible job opportunity, and most described substantial improvements in their economic wellbeing with time in Canada and experience in the sex industry. Indeed, most participants valued the steady income (median: $3200/month) and flexibility that sex work provided:

[When I first arrived] I could only find jobs that don’t require a lot of English…[such as] a Chinese restaurant or factory… it pays only 8 bucks an hour…you start around 10 or 11 am, and finish work at night…the working hours were very inconvenient for me to look after my child. **The work I’m doing right now offers me more flexibility to take care of my child. A lot of the workers are like me.**

[QE34, worker, 6y in Canada]

#### Policing and language barriers to health and human rights during long-term settlement

Following entry into sex work and concomitant improvements in economic security, participants routinely identified the legal and social ramifications of stressful interactions with police and language barriers to be the most crucial determinants of their health and safety during settlement in Canada.

“We’re afraid of leaving a bad record”: Fear of sex work disclosure or legal consequences of police interactions: AESHA data indicate that im/migrants in Vancouver’s sex industry experience high levels of criminalization, including police arrest or harassment (e.g., raids, fines, detained, property taken) (*n =* 42, 21.2%). Interviewees expressed deep concern regarding the legal and social ramifications of this. Due to stigma and legal concerns, most undertook great efforts to conceal their work from family (e.g., spouses, children), and feared that police visits or criminal proceedings could result in public disclosure of sex work involvement. For example, the stigma, family conflict, and exposure an arrest could entail (e.g., having one’s name or photograph in the news) was a serious source of stress for most participants:

I worry about being found out by my family. It would have a **psychological impact on my daughter and cause conflicts** between my husband and I…**If I do indeed get caught by the police, my work will get exposed, and the thought of that gives me a lot of stress**.

[QE44, worker, 1y in Canada]

Most working women are afraid of getting caught [by police] when they are engaging in sexual activity in the room. They are also **afraid of the police telling their families, relatives, and friends** about their job. **Having their name exposed publicly and facing a criminal charge** are also concerns.

[QE37, manager/worker, 10y in Canada]

**I am the most afraid of having my name exposed publicly.** I am also afraid of criminal charges and **being looked down by my child.**

[QE36, manager/worker, 10y in Canada]

Whereas most interviewees had legal immigration status in Canada (i.e., citizenship, permanent residence, or a temporary work permit), those who were not naturalized citizens worried that interacting with law enforcement could jeopardize this status or their future citizenship prospects, particularly in light of immigration policies explicitly prohibiting sex industry work for work permit holders:

[What do most working women worry about when seeing police?] **We are afraid of leaving a bad record, and we are afraid our identity or names would be exposed**…I’d be **afraid of this affecting my immigration status, and losing it.**

[QE41, worker, 2y in Canada]

The new immigrants are **afraid of their status being revoked or taken away**. They are also afraid of **not being able to apply for citizenship**.

[QE37, manager/worker, 10y in Canada]

#### Discriminatory and racialized treatment by law enforcement

Some participants attributed the heightened scrutiny of law enforcement towards Chinese-run and staffed establishments to ethnic discrimination, noting unexplained documentation checks and raids to disproportionately target Chinese businesses. Unexplained police visits and document checks, combined with discriminatory and disrespectful treatment, fostered deep mistrust between venue personnel and law enforcement:

**Immigrants like us rarely have contact with police, so we are all quite nervous.** This is only natural…**we should not have to interact with the police much here, unless we are in danger, or if we commit crimes**. So now, even when we didn’t do anything, there are police here asking us many questions, which is very strange, and **it is very uncomfortable**. Especially their attitudes when they question us, **it’s as if we are doing something wrong**.

[QE29, manager, 3y in Canada]

The police are driving me crazy. Why do they come here? They scrutinize us so closely…**it’s discrimination**…Sometimes we can understand why we were treated this way. Even though we are Canadian citizens, **we do not feel we are treated equally as local people.** It’s really difficult for foreigners to survive.

[QE30, manager, 10y in Canada]

#### Criminalization and policing as barriers to im/migrant workers’ health, safety, and access to support

Rather than perceiving the role of law enforcement as protective, the stress created by police presence was framed as yet another risk to be managed by im/migrant workers and managers. As one participant explained how undue stress regarding law enforcement raids could compete with other priorities, such as sexual and mental health:

Many who work in this trade feel burdened because they have to think about possibly contracting HIV, other STIs, worrying about their own health, being found out by the police, and their own emotional suffering–altogether, it’s a large burden. However, if sex work is legalized, then their burden would decrease quite a bit–since this is a job like any other job, only they would have to take care of their health. **They would not have to worry about much else, such as hiding once the police enter the parlour**.

[QE15, worker, 10y in Canada]

Moreover, police raids and inspections often led managers to avoid anything that could be considered evidence of sex work, such as discussing HIV/STI prevention with workers or permitting large quantities of condoms onsite. As the following worker noted how fear of police restricted opportunities for managers to promote occupational health and safety:

The manager just tells us to be careful…but **she doesn’t have the ability to do too much.** She’s also afraid of the police coming here and knowing that we’re doing this work. It would shut down her parlour.

[QE28, worker, 3y in Canada]

Interactions with police were particularly distressing in light of language barriers (interpreters were rarely provided) and the value migrants placed on staying out of the purview of law enforcement. A number of interviewees described themselves and their co-workers as “good immigrants” who went to great lengths to avoid what they perceived to be illicit activities (e.g., drug use). Participants recognized that the combination of their status as im/migrants, language barriers and inadequate access to legal information rendered them poorly equipped to handle police inquiries when they did occur, a further motivator to avoid police to ensure their good standing in Canada:

We don’t want to cause trouble. That’s what Chinese people are used to, living in a foreign country; **we very carefully protect ourselves, and try to not to commit any violations**, because if we were to bring ourselves any hassle, **our English isn’t good, and we don’t really understand too much about the law**…so of course, **the further we stay away, the more careful we can be, the better.**

[QE18, manager/worker, 9y in Canada]

The precautions im/migrants took to avoid interacting with law enforcement often directly jeopardized their health and safety. This often took the form of taking extra care to ensure client satisfaction, as one participant expressed, “We can’t offend any client…we don’t want to cause trouble” (QE18). Some workers described the need to be more “accommodating” to clients’ demands (e.g., for activities which could place workers at increased risk of STIs or sexual assault):

Sometimes they will get angry and I couldn’t really say anything because I don’t know English, right? When he gets angry and says that I did something poorly… **you just have to be accommodating.**

[QE27, worker, 3y in Canada]

Fear of criminalization intersected with concerns of prejudicial treatment by police to pose barriers to seeking assistance or reporting cases of violence. Im/migrants in AESHA reported high levels of client-perpetrated physical and sexual violence (*n =* 33, 16.7%). Yet, in qualitative interviews, most were reluctant to report violence to law enforcement due to the expectation that their concerns would not be taken seriously and fear of criminal charges. Interviewees were highly critical of this unequal access to legal protections:

People have inherent prejudice against this occupation…I feel that when I’m treated unfairly by the clients, I have no place to report unfair treatment to the police or any authority, or to seek protection.

[QE44, worker, 1y in Canada]

If anything happened, I am not willing to call the police…Why is it that other people, when they have anything dangerous come up in their work, they can call the police? Why don’t we receive the same kind of protection?…**if the women had this kind of protection, then people out there also wouldn’t commit these offences.**

[QE28, worker, 3y in Canada]

#### Language barriers to occupational health and safety

Although most interview participants perceived that their English was sufficient to “learn the basics about a client”, those who lacked English fluency identified language barriers to dealing with difficult clients. Some clients tried to take advantage of workers’ limited English skills to attempt to coerce them into unsafe or unwanted sexual activities, and in some cases, threatened to call the police if workers did not acquiesce to their demands. Criminalization and language barriers intersected to jointly undermine workers’ access to workplace health and safety, particularly for recent arrivals:

There was a client who asked for service without condom and **tried to take advantage of my poor English skill.** After I refused to provide service without condom, he intentionally broke the condom, but fortunately I found out. So I figured to provide the service again, and asked him to use a new condom–he got mad…**He also threatened to call the police**…he was trying to threaten to report me.

[QE44, worker, 1y in Canada]

He secretly pulled the condom [off] but I didn’t realize…of course, he just pretended like he didn’t know. I believe that he did it on purpose. There’s no way that he didn’t know, because when he started there was a condom on.

[QE28, worker, 3y in Canada]

Limited English fluency also represented a powerful barrier to accessing sexual health, safety, or legal supports. Most workers and managers were unaware of local organizations or resources offering health, social, or legal support for sex workers. Many participants noted the inaccessibility of resources offered in English only (e.g., websites with descriptions of unsafe clients; information regarding workers’ rights). Poor access and difficulties understanding legal information made it particularly difficult for im/migrants to ascertain when or whom to contact in cases where assistance was desired:

Because of the English language barrier, I don’t understand too much about it [prostitution laws and regulations]. What I have is not a detailed understanding.

[QE13, manager/worker, 2y in Canada]

[Do you know of any social services that can help you in case of client violations?] No, I don’t…**There are still many laws in Canada that I am not quite familiar with. Most of the times, they speak English so I can’t understand it.**

[QE12, worker, 7y in Canada]

## Discussion

In this study, the narratives of im/migrant workers and managers in the indoor sex industry revealed a diversity of pathways into sex industry work upon arrival to Canada, including language barriers to conventional labour markets, and the higher pay and relative flexibility (e.g. scheduling) of sex industry work. Once engaged in sex work, fear associated with police raids (e.g., immigration concerns, sex work disclosure) and language barriers to sexual negotiation and access to health, social and legal supports posed pervasive challenges to health, safety and human rights during settlement in Canada. Importantly, all participants migrated to Canada voluntarily, and none identified as having been trafficked, countering dominant portrayals of migrant sex workers as trafficking victims.

As previous research has shown that experiences related to both im/migration and sex work can shape health outcomes, our analysis focused on the intersection between these two dimensions. Whereas much previous research has shown that im/migrant sex workers often face very different health outcomes and behavioural risks than non-migrant sex workers[6, 52, 53], few studies have examined the underlying social and structural factors that may explain these differences (e.g., working conditions, racialization), or how these underlying determinants may change across the stages and duration of im/migration. This study uniquely addresses this gap by describing the evolving social and structural determinants of health and safety among im/migrants in the indoor sex industry across the arrival and settlement process in Vancouver, Canada, including experiences related to both sex work and im/migration.

Findings of this study highlight the pervasive roles of criminalization, language barriers, and intersecting concerns of stigma and discrimination in shaping the occupational health, safety, and wellbeing of im/migrants in Canada’s indoor sex industry. Moving beyond individually focused approaches to understanding im/migrants and sex workers’ health and wellbeing, our findings illustrate how large-scale forces such as sex work criminalization, restrictive im/migration policies, economic disadvantage and racialization jointly shape the health, safety and human rights of im/migrant sex workers, and highlight the pervasive structural vulnerability experienced by this disadvantaged group[41–43].

Whereas migrants’ initial concerns upon arrival in Canada were primarily economic, fear of negative consequences of interacting with police and language barriers were framed as the most salient influences on im/migrants’ health and safety during long-term settlement in Canada. Although previous work has identified economic insecurity, language barriers, and limited employment opportunities as key concerns for migrant workers in destination settings,[54–57] much less research has acknowledged the ways in which the racialized enforcement of criminal laws (i.e., targeted surveillance of massage parlours) affects the health and wellbeing of im/migrant women who do sex work.

In contrast to previous research attributing changes in migrant health over time largely to the acculturation process[36, 37], our findings suggest that migrant health inequalities may often be more broadly linked to features of the social and structural environment, such as discriminatory, gendered and racialized law enforcement and immigration surveillance targeting massage parlours.[58] The fear of having sex work activities disclosed to family or the public, discriminatory treatment by police, and fear of criminal charges or loss of immigration status often caused workers and managers to go to great lengths to avoid interacting with police; for example, reluctance to call the police due to fear of criminalization was perceived to provide clients with leverage to abuse workers with impunity. Previous evidence indicates that as a result of economic insecurity, precarious legal status and lack of political and social capital, im/migrant workers are more likely to work under unsafe conditions and to experience discriminatory practices and legal/policy environments that may undermine health[59] and make it unlikely that workplace abuses will be reported.[2, 60] Despite their stated intention of protecting sex workers and trafficked individuals, the ramifications of police inspections and raids for im/migrant workers in this study included enhanced marginalization and insecurity. Fear of criminalization intersected with language barriers and stigma to undermine health and well-being, such as by discouraging im/migrant women from reporting crimes to police. This affirms the findings of studies indicating the harms of criminalization for sex workers generally[61], and provides key evidence contextualizing quantitative data indicating the links between criminalization and sexual risk and violence among im/migrant women in sex work.[62]

These findings are of particular concern given newly introduced laws that criminalize new aspects of sex work (*The Protection of Exploited Persons and Communities Act*) in Canada. This new legislation was introduced despite a 2013 Supreme Court ruling striking down Canada’s prostitution laws as unconstitutional, with substantial evidence that this approach is likely to reproduce similar harms (e.g., lack of access to safe workspaces)[61, 63] and to push im/migrant sex workers further underground and away from health and social supports.[49, 63] Previous research internationally has shown that criminal laws surrounding the sex industry are often underpinned by concerns regarding trafficking and often contribute to enforcement efforts that fail to advance the health and human rights of sex workers. For example, punitive measures targeting the sex industry are often implemented in response, including police crackdowns, raids, and rescue operations in sex work venues.[64, 65] In Canada and elsewhere, current legislative frameworks criminalizing aspects of the sex industry (e.g., solicitation, advertisement, purchasing) and their enforcement have been shown to contribute to unintended harms that exacerbate health inequities and human rights violations–for example, by displacing workers to isolated, unsafe work environments; undermining their access to healthcare; and increasing their vulnerability to police abuses.[61, 65–67] Findings of this study extend this work by showing how criminalized sex work legislative frameworks and their harms may be uniquely exacerbated for im/migrant sex workers in Canada. These results indicate the critical need to shift to human rights-based, decriminalized models that avoid conflation of sex work and trafficking to promote the health and safety of im/migrants working in the sex industry.

### Limitations and future directions

To address the challenges involved in research and service provision involving highly criminalized and often hidden populations, including im/migrants and sex workers (e.g., difficulties in access, establishing rapport, stigma), outreach and interviews were conducted by a multicultural and multilingual team with deep sex work community experience. Additionally, through our longitudinal study design, we engage in weekly outreach to develop rapport and meaningful, long-term relationships with im/migrant sex workers and managers in the community; third, our team is highly trained in de-stigmatizing and rights-based approaches to supporting im/migrant sex workers’ health and wellbeing.

Despite our best efforts, our study does not reflect the full diversity of more marginalized im/migrants who do sex work, such as undocumented individuals. Further studies designed *a priori* to elucidate evolving determinants of health across the migration process (e.g., pre-migration, transit, arrival, long-term settlement) remain needed. Comparative and cross-national studies are also recommended to investigate the health effects of im/migration across different communities of origin, transit, and destination. Further research investigating social determinants of migrant health remains needed to elucidate the pathways leading to changes in health across the migration process, particularly among highly marginalized subgroups (e.g., undocumented migrants).

Future mixed-methods, qualitative, and social epidemiological research conducted in collaboration with im/migrant sex workers is recommended to inform the design and evaluate the impacts of models of health and social service provision for im/migrants in the sex industry. Efforts to evaluate the impacts of different intervention approaches on access to health services, safety, and legal information for im/migrant sex workers are particularly recommended. For example, Vancouver is home to several innovative community-based efforts, such as multilingual and culturally safe outreach to indoor and online spaces (e.g., program operated by SWAN, Supporting Women’s Alternatives Network, in Vancouver); the provision of written information regarding legal rights (e.g., ‘rights cards’ educating workers about relevant legislation and legal rights, in various languages); and safer indoor workplace interventions (e.g., provision of security, guest policies). Research evaluating these models, developed and led in partnership with im/migrant and sex work communities, is recommended to inform the most effective strategies.

### Conclusions

This analysis of social and structural determinants of health and safety among im/migrants in the indoor sex industry in Canada revealed a diversity of pathways into sex industry work upon arrival to Canada, including language barriers to conventional labour markets, and the higher pay and relative flexibility (e.g. scheduling) of sex work. In the long-term settlement phase, fear associated with police raids (e.g., immigration concerns, sex work disclosure) and language barriers to sexual negotiation and access to health/social services and legal information posed serious challenges to im/migrants’ health, safety and human rights. This study highlights the critical influences of the intersection between criminalization and language barriers for the occupational health and safety of im/migrants in Canada’s sex industry, and highlights the importance of structural interventions and policy reforms to promote health and wellbeing across the arrival and settlement process. Rather than attributing the disparities in wellbeing and access to health/social services access to the acculturation process, it is crucial that more complex structural analyses inform the delivery of these services, including racial discrimination, criminalization of sex work, as well as the conflation of migration, sex work, and trafficking. While further research is needed, it is clear from our results that a rights-driven approach to development of law and policy is necessary to support health equity for im/migrant women who do sex work.
